# Decoding Preeclampsia and Obesity: The Role of Immune Responses and Natural Product Interventions

**DOI:** 10.2174/0118715303461365260414142741

**Published:** 2026-04-29

**Authors:** Xi Long, Wene Liu, Jie Lin, Qinghua Peng

**Affiliations:** 1 College of Chinese Medicine, Hunan University of Chinese Medicine, Changsha, Hunan, China;; 2 Department of Obstetrics & Gynecology, First Affiliated Hospital of Hunan University of Chinese Medicine, Changsha, Hunan, China

**Keywords:** Preeclampsia, obesity, characteristic genes, natural products, bioinformatics, mendelian randomization

## Abstract

**Introduction:**

Preeclampsia (PE) is a pregnancy-specific disorder with high cerebrocardiovascular risk. Obesity increases PE risk, but current monotherapy remains insufficient. Shared mechanisms and natural product-based interventions for PE regarding obesity are urgently needed.

**Methods:**

Mendelian Randomization (MR) was used to investigate the causal relationship between PE and obesity, and datasets from GEO were analyzed. Differential expression, enrichment analysis, PPI network, and machine learning (random forests, SVM) were applied to identify characteristic shared genes, which were validated using external datasets. ROC curves assessed diagnostic efficacy. CIBERSORT analyzed immune infiltration. ITGB5 expression was further evaluated using the HPA database. Natural products targeting characteristic genes were predicted, followed by molecular docking and dynamics analysis.

**Results:**

MR confirmed obesity as a causal risk factor for PE. GSE96985 and GSE2508 were core datasets. Sixty-nine common DEGs were enriched in muscle cell cytoskeleton pathways. Twenty-five characteristic genes were screened; six genes showed significant diagnostic efficacy. Immune infiltration suggested CD8+ T cells as a shared immune cell type. ITGB5 was highlighted. HPA confirmed high expression in the placenta and adipose tissue. Puerarin formed a stable complex with ITGB5 (docking energy: -5.92 kJ/mol), supported by molecular dynamics.

**Discussion:**

ITGB5 may link PE and obesity *via* immune-metabolic crosstalk, with CD8+ T cell dysregulation potentially contributing to inflammation, oxidative stress, and endothelial dysfunction. Puerarin emerges as a promising natural agent.

**Conclusion:**

These findings suggest that ITGB5 is a candidate shared gene in PE-obesity comorbidity, and that puerarin is a promising natural agent. Given the bioinformatic nature, these conclusions are hypothesis-generating.

## 1 INTRODUCTION

Preeclampsia (PE) is a pregnancy-specific multisystem disorder marked by the onset of hypertension and proteinuria after 20 weeks of gestation, affecting 2%–5% of pregnancies globally and constituting a major cause of maternal and perinatal morbidity [1]. The pathogenesis of PE is attributed to placental dysfunction, endothelial damage, and systemic inflammation, with clinical manifestations ranging from mild hypertension to severe eclampsia [2]. PE is linked to long-term cardiovascular risks, including hypertension and endothelial dysfunction. Obesity, defined by a Body Mass Index (BMI) of 30 kg/m^2^ or greater, has reached pandemic proportions, impacting over 650 million adults worldwide [3]. Currently, the prevalence of obesity among women of childbearing age is 31.8% [4]. Obesity, a metabolic disorder, is characterized by chronic low-grade inflammation, insulin resistance, and dysregulation of adipokines, predisposing individuals to cardiovascular and metabolic diseases [5]. Specifically, obesity exacerbates cardiovascular strain by increasing cardiac workload and vascular remodeling. Both PE and obesity share overlapping risk factors, including dyslipidemia, oxidative stress, and pro-inflammatory states, indicating potential shared mechanisms of action.

Research has indicated that women with obesity face a two- to three-fold elevated risk of developing PE, with the severity of the condition being positively correlated with BMI [6]. This risk extends beyond pregnancy, as obesity-related PE significantly elevates the lifetime risk of cardiovascular morbidity. Cohort studies have revealed that excessive adiposity exacerbates placental hypoxia, intensifies inflammatory responses, such as increased levels of Tumor Necrosis Factor-alpha (TNF-α) and interleukin-6 (IL-6), and impairs trophoblast invasion, which are key features of PE pathogenesis [7]. Endothelial dysfunction contributes to the development of PE and cardiovascular damage. Mechanistic investigations using High-Fat Diet (HFD) mouse models have demonstrated the emergence of PE-like phenotypes, including hypertension and proteinuria. Despite these advancements, the precise interactions between metabolic dysfunction and placental pathology remain insufficiently understood, particularly regarding the shared signaling pathways that could be targeted for therapeutic intervention.

Bioinformatics analyses have enhanced our understanding of the intricate pathophysiological mechanisms underlying disease comorbidities. Researchers have identified key molecular pathways shared by seemingly disparate diseases by integrating datasets from the Gene Expression Omnibus (GEO) with computational methods, such as differential gene expression analysis and functional enrichment. Recent studies have successfully employed these approaches to explore comorbidities such as psoriasis and obesity [8] or premature ovarian failure and dry eye disease [9], demonstrating the utility of bioinformatics in uncovering shared pathophysiological mechanisms. However, despite the well-established epidemiological link between PE and obesity, the application of bioinformatics to elucidate their shared molecular mechanisms has not been explored. Existing studies have focused on individual aspects of each condition, such as inflammatory pathways in obesity or angiogenic imbalance in PE, without systematically investigating the overlapping gene signatures and regulatory networks that drive comorbidities. Although emerging evidence suggests the potential of natural compounds in treating PE [10, 11], few studies have integrated bioinformatics-based drug prediction to identify therapeutic agents that could simultaneously target both conditions. Recent studies have advanced our understanding of the obesity-PE link, demonstrating genetic correlations and bidirectional causality between BMI and PE [12]. Another study has revealed causal associations between multiple adiposity measures and PE risk using Mendelian Randomization (MR)[13]. However, these studies have limitations; they relied on Genome-Wide Association Study (GWAS) summary data without integrating tissue-specific expression profiles or systematic multi-dataset analysis, and none extended their findings to predict therapeutic agents targeting shared pathways.

This study aimed to address these gaps by analyzing GEO datasets related to PE and obesity to identify shared molecular signatures and potential therapeutic targets. Specifically, we employed an integrated bioinformatics approach combining differential expression analysis, weighted gene co-expression network analysis, and machine learning algorithms to identify hub genes common to both conditions. Additionally, MR was used to assess causal relationships between identified genes and disease outcomes, providing genetic evidence that complements the bioinformatics findings. Finally, the authors investigated bioactive natural compounds that may simultaneously modulate these pathological pathways using molecular docking and drug prediction analyses. This study represents the first systematic integration of bioinformatics, Mendelian Randomization (MR), and natural compound prediction aimed at elucidating the shared mechanisms and therapeutic potential for Preeclampsia (PE)-obesity comorbidity. The comprehensive workflow is detailed in the graphical abstract.

## 2 MATERIALS AND METHODS

### 2.1 Mendelian Randomization

To enhance the robustness of this study, MR was performed to investigate the causal relationship between PE and obesity. GWAS summary-level data for PE and obesity were retrieved from the OpenGWAS database (https://gwas.mrcieu.ac.uk/). The detailed characteristics of all datasets are summarized in Table **1**. Single-Nucleotide Polymorphisms (SNPs) associated with the two phenotypes were initially extracted with a genome-wide significance threshold of *p* < 5 × 10^−5^ as candidate Instrumental Variables (IVs). To guarantee the independence of IVs, Linkage Disequilibrium (LD) clumping was conducted using strict parameters (r^2^ = 0.001, physical distance = 10,000 kb) to eliminate redundant SNPs. After rigorous screening, 100 independent and valid SNPs were retained as the final IVs for obesity (exposure) and PE (outcome). The strength of the final IVs was evaluated using the F-statistic, with a mean F-statistic of 10 for both phenotypes, confirming the absence of weak instrument bias in this study.

Five analytical approaches were applied for MR analysis: the inverse variance weighted method was used as the primary strategy to estimate the causal effect, while MR-Egger regression, weighted median, simple mode, and weighted mode were adopted as complementary methods for cross-verification. A two-sided *p* < 0.05 was defined as the statistical significance threshold for the causal association between exposure and outcome. Multiple sensitivity analyses were performed to assess heterogeneity and horizontal pleiotropy of IVs. Cochran’s Q test revealed no significant heterogeneity among the included IVs (Q = 75.851, *p* = 0.296). The MR-Egger intercept test indicated no evidence of directional horizontal pleiotropy (intercept = –5.729e-05, *p* = 0.262). Additionally, the MR-PRESSO global test failed to detect significant outlier SNPs (test statistic = 79.191, *p* = 0.314), further verifying the absence of heterogeneity induced by abnormal variants and supporting the robustness of our MR estimates.

### 2.2 Data Source

Datasets on PE and obesity were obtained *via* a systematic search of the GEO database (https://www.ncbi.nlm.nih.gov/gds/). The inclusion criteria were as follows: (i) Each group must have a sample size of at least three, (ii) the samples from both normal and control groups must be included, and (iii) the dataset must provide raw, comprehensive data that can be further exported for analysis. All GEO datasets were downloaded in MINiML format, which contains complete platform, sample, and series record information. For datasets that were not already log2-transformed, we applied a log2 transformation. For datasets that had not been normalized, we performed quantile normalization using the normalize. The quantiles function from the preprocessCore R package. Probe IDs were converted to gene symbols based on the corresponding platform annotation files. Probes matching multiple genes were excluded, and for multiple probes mapping to the same gene, the average expression value was calculated. For datasets with multiple batches on the same platform, batch effects were removed using the removeBatchEffect function from the limma R package. When integrating multiple datasets or analyzing data from different platforms, we first identified common gene symbols across datasets, then treated each dataset or platform as a separate batch and applied the removeBatchEffect function to correct for batch effects. The PE-related dataset GSE96985 and the obesity-related dataset GSE2508 were selected for data analysis. To reduce errors associated with using a single dataset, additional PE-related datasets GSE60438 and GSE25906, and the obesity-related dataset GSE151839, were selected to validate the results of subsequent analyses. Specific information regarding the dataset is presented in Table **2**.

### 2.3 Identification of Differentially Expressed Genes (DEGs)

After screening the GEO dataset, the matrix files obtained from the platform were preprocessed, and the probes were matched to their gene symbols using the platform's annotation files. Finally, gene matrices were obtained with row names as gene symbols and column names as sample names for subsequent analyses. DEGs associated with PE and obesity were identified using the limma package in R software. Subsequently, normalization and analysis were performed using the limma package, with a False Discovery Rate (FDR) threshold of < 0.05 and a Fold Change (FC) criterion of > 1.5 This yielded a set of DEGs encompassing both upregulated and downregulated genes.

### 2.4 Identification and Analysis of Shared Genes in PE-obesity Comorbidity

A Venn diagram was used to identify the intersection of pre-screened DEGs (FDR < 0.05 and |log2FC| > log2(1.5)) for PE and obesity, thereby identifying the shared genes associated with both conditions. To further elucidate signaling pathways and biological processes implicated in the enrichment of these shared genes. Kyoto Encyclopedia of Genes and Genomes (KEGG) enrichment analysis and Gene Ontology (GO) functional annotation were performed using the “clusterProfiler” package in R language. GO function annotations cover Biological Processes (BP), Molecular Functions (MF), and Cellular Components (CC), which can be analyzed to obtain a more comprehensive understanding of gene function. Results were considered significant at a threshold of *p* < 0.05. The KEGG enrichment analysis provided enhanced insights into the signaling pathways in which the shared genes were enriched.

### 2.5 Protein-Protein Interaction (PPI) Network Construction and Modular Analysis

The STRING database (https://string-db.org), a comprehensive online resource for retrieving interacting genes, was employed to construct a PPI network, with the species parameter set to “*Homo sapiens.*” The constructed PPI network was imported into Cytoscape (http://www.cytoscape. org; version 3.8.2) for analysis and visualization. The MCODE plugin was used to extract closely interacting subnetworks to identify key functional modules within the network. The analysis was conducted using the following parameters: Degree cutoff = 2, maximum depth = 100, node score cutoff = 0.2, and K-core = 2. Additionally, PPI genes were evaluated using the degree-value algorithm in the CytoHubba plugin to assess gene importance and identify hub genes within the network. The analysis employed MCC, DMNC, and Degree values as reference standards,

### 2.6 Support Vector Machine-recursive Feature Elimination (SVM-RFE) and Random Forest Analysis

To further investigate the diagnostic potential of the shared genes, a predictive machine learning model was developed. Specifically, the advanced SVM-RFE algorithm from the e1071 package was used to construct the model. This analysis provided a more nuanced understanding of these genes. Additionally, the analysis was implemented using the randomForest package in R, optimizing parameters through 10-fold cross-validation. The Gini importance metric was used to rank genes according to their contribution to classification accuracy, with higher scores indicating greater discriminative power. Genes whose mean decrease in Gini exceeded the threshold (cutoff = 2.0) were retained as candidate biomarkers for subsequent validation.

### 2.7 Identification and Analysis of Characteristic Shared Genes

A Venn diagram (https://jvenn.toulouse.sra.fr/app/example.html) was used to identify the intersection of genes from PPI modular and machine analyses, thereby facilitating the identification of characteristic genes shared between PE and obesity. Subsequently, KEGG enrichment analysis and GO functional annotation were performed using the “clusterProfiler” package in R language. The results were screened at *the *p* < 0.05 level*. Next, GeneMANIA (https://genemania.org/) was used to construct co-expression networks for the identified characteristic hub genes.

### 2.8 Transcription Factors (TFs) Analysis and Construction of Regulatory Network

The Transcriptional Regulatory Relationships Unraveled by Sentence-based Text mining (TRRUST) database was used to predict transcriptional regulatory networks. It encompasses target genes associated with TFs and the regulatory relationships between TFs. TRRUST currently includes two species: human and mouse, with 8,444 and 6,552 TF-target regulatory relationships, respectively, for 800 human and 828 mouse TFs. TFs regulating characteristic genes were identified using the TRRUST database, with statistical significance determined at an adjusted *p* < 0.05. Subsequently, the expression levels of these TFs were validated using a t-test.

### 2.9 Validation of Characteristic Shared Genes

To further elucidate the significance of the characteristic genes identified through the analysis, validation was conducted using an independent dataset in R. The expression levels of the identified genes between the control and disease groups were compared using the Wilcoxon rank-sum test, with *p* < 0.05 considered statistically significant. Afterward, the expression patterns of the characteristic shared genes were analyzed and visualized.

### 2.10 Receiver Operating Characteristic (ROC) Curve and Immune Infiltrate Analysis

The ROC curve analysis was employed to develop models for evaluating the predictive performance of outcomes and the diagnostic value of a particular factor. Since these genes were selected based on differential expression and functional analyses in the discovery datasets (GSE96985 for PE and GSE2508 for obesity), we performed ROC analysis directly in two independent external validation datasets (GSE25906 for PE and GSE151839 for obesity) to assess their diagnostic performance without overfitting bias. The R package pROC was used to perform the ROC analysis and obtain the AUC. An area under the curve (AUC) value > 0.5 and ~1 indicates high diagnostic accuracy. AUC values between 0.5 and 0.7 indicate low accuracy, 0.7 to 0.9 moderate accuracy, and > 0.9 high accuracy. Specifically, the patient's disease status, along with gene expression, was obtained, and the AUC and Confidence Intervals (CIs) were evaluated to assess the diagnostic value of the genes in the disease.

CIBERSORTx, an analytical tool developed by the Alizadeh Lab and the Newman Lab, was used to impute gene expression profiles and estimate the abundance of 22 distinct immune cell types in a mixed cell population [14]. The CIBERSORT method was performed using the R software packages e1071 (required for the CIBERSORT core algorithm) and ggplot2. Immune cell fractions were inferred using the LM22 signature matrix with 1,000 permutations to generate statistically significant deconvolution results. Only samples with a CIBERSORT *p* < 0.05 were retained for downstream analysis. To assess the relationship between characteristic genes and immune cell infiltration in the tissue microenvironment, a Mantel test was performed using the vegan R package. The Mantel statistic (r) was computed as Pearson's correlation between distance matrices derived from gene expression and immune cell proportions. Statistical significance was determined using 999 permutations of the distance matrix. *P*-values were adjusted for multiple comparisons using the Benjamini-Hochberg FDR method. The correlation coefficients and significance levels were visualized using the ggstatsplot R package.

### 2.11 Gene Set Enrichment Analysis (GSEA) and Human Protein Atlas (HPA) Database Analysis

To elucidate the functional dynamics and pathway alterations of the most characteristic gene identified from the analyses, GSEA, a method used to assess the significance of genomic changes between two biological states, was used to select the main enrichment functions. GSEA soft bases on MSigDB were used to annotate genomes, and significance was set at *p* < 0.05. These analyses helped identify the functional and pathway changes in these genes. The biological processes and pathways potentially associated with each gene were elucidated by scoring and visualizing them using R, thereby providing a more nuanced understanding of their roles in disease mechanisms.

The expression profiles of the hub gene in human tissues were retrieved from the HPA database (https://www.proteinatlas.org/). Immunohistochemistry (IHC) results of characteristic genes shared between the placenta and adipose tissue were analyzed for staining level, intensity, positive cell ratio, and subcellular localization. Normalized RNA-seq data (nTPM) were used to compare integrin β5 (ITGB5) expression in the placenta, adipose tissue, and other normal human tissues.

### 2.12 Drug Forecasting and Analysis

The HIT2.0 (http://www.badd-cao.net:2345/) platform was used to perform reverse prediction based on hub genes and identify potentially active Traditional Chinese Medicine (TCM) natural products associated with these characteristic shared genes. Subsequently, additional information, such as Lipinski's five-fold rule and gastrointestinal absorption of natural products, was retrieved using SwissAMDE (http://www.swissadme.ch/). Following the identification of the natural product, molecular docking analysis was performed between the hub genes and the natural product. The 3D structures of the proteins (PDB format) were downloaded from the PDB database (https://www.rcsb.org/), and the *mol2 format of the analyzed structures of the corresponding natural products was collected using TCMSP (https://old.tcmsp-e.com/tcmsp.php). Docking was conducted after converting the compound and target protein to the *pdbqt format using AutoDock. The docking results were imported into PyMOL for visualization [15].

Subsequently, to further investigate the interactions between the target protein and the small molecule ligand, molecular dynamics simulations were performed using GROMACS 2022. Force field parameters for the protein were generated using the pdb2gmx tool with the AMBER14SB force field. The ligand topology was prepared using sobtop_1.0 software under the GAFF2 force field, with partial charges assigned *via* the RESP method. The system was solvated in a 1 nm cubic box using the TIP3P water model, and Na^+^and Cl^−^ions were added to a concentration of 0.15 M to maintain electroneutrality. Long-range electrostatic interactions were treated using the particle mesh Ewald method, and bond constraints were applied using the LINCS algorithm. Prior to simulation, energy minimization was performed in three stages: first with the solute constrained, then with ions constrained, and finally without constraints. Simulations were conducted under the NPT ensemble at 310 K (Nosé–Hoover thermostat) and 1 bar (Parrinello–Rahman barostat) for 100 ns with a 2 fs integration time step. Trajectory analysis, including Root Mean Square Deviation (RMSD), Root Mean Square Fluctuation (RMSF), hydrogen bond number, radius of gyration (Rg), and Solvent-Accessible Surface Area (SASA), was performed using GROMACS tools to evaluate system stability, structural flexibility, and solvation effects.

## 3 RESULTS

### 3.1 Mendelian Randomization

**Fig. (1):**
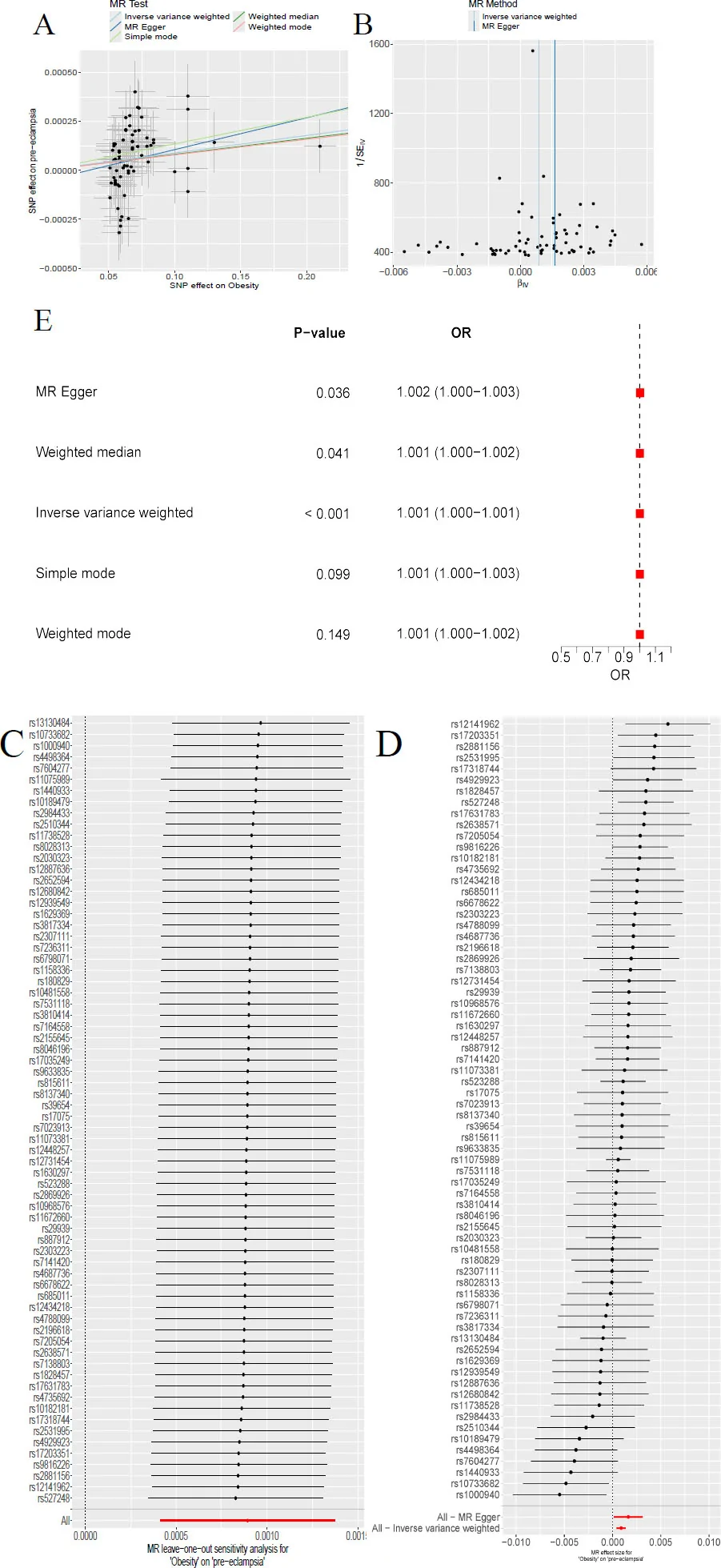
Plots of MR estimates illustrating the causal effects of obesity and PE. (**A**) Scatter plot of SNPs associated with obesity and their corresponding risk of PE. (**B**) Funnel plot assessing the pleiotropy of the observed causal associations between obesity and PE. (**C**) Leave-one-out plot of SNPs associated with obesity and their impact on PE. (**D**) MR effect size of SNPs associated with obesity and their associated risk of PE. (**E**) MR estimate of the causal relationship between obesity and PE. CI, confidence interval; OR, odds ratio.

We investigated the relationship between PE and obesity using MR analysis. The MR analysis results demonstrated a significant positive causal relationship between obesity and PE (*p* < 0.001; Odds Ratio [OR] = 1.001; 95% CI, 1.000-1.001). The forest plot of SNPs further confirmed that obesity is an exposure factor for PE development. The leave-one-out sensitivity analysis revealed that the effect sizes of the included IVs were similar to the overall effect size, affirming the robustness of the analysis (Fig. **1**).

### 3.2 Identification of DEGs

Differential expression analysis was conducted using the GSE96985 and GSE2508 datasets. This analysis identified 2,794 DEGs associated with PE and 601 associated with obesity. Specifically, in the PE dataset, 2,794 genes were differentially expressed, with 1,153 upregulated and 1,641 downregulated genes. In the obesity dataset, 601 genes were differentially expressed, comprising 440 upregulated and 161 downregulated genes. These findings are illustrated in Fig. (**2**).

### 3.3 Identification and Analysis of Shared Genes in PE-obesity Comorbidity

Following the intersection of the DEGs obtained through the above analysis, the study identified 69 shared genes between PE and obesity. Subsequently, an enrichment analysis of these shared genes was performed. In KEGG enrichment results, 22 relevant pathways were identified, primarily involving the cytoskeleton in muscle cells and osteoclast differentiation. Gene Ontology (GO) enrichment analysis identified 503 significant terms across the Biological Process (BP), Cellular Component (CC), and Molecular Function (MF) categories associated with PE-obesity comorbidity. As illustrated in Fig. (**3**), the most prominent enrichments included extracellular matrix organization (BP), collagen-containing extracellular matrix (CC), and integrin binding (MF).

### 3.4 PPI Network Construction and Modular Analysis

**Fig. (4):**
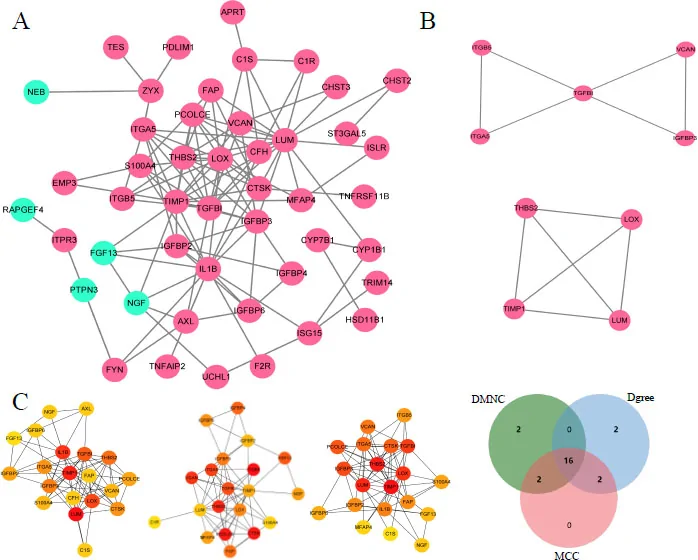
PPI network analysis. (**A**) PPI networks from String10.0. Red nodes indicate positive correlation with disease, and green nodes indicate the opposite. (**B**) Clusters 1 and 2 from the MCODE analysis of characteristic shared genes. (**C**) Identification of characteristic shared genes using CytoHubba of MCC, DMNC, and Degree ranking methods.

The characteristic shared genes were imported into the STRING10.0 database (https://string-db.org/) with the species parameter set to “*Homo sapiens*” to obtain the PPI network. The characteristic shared genes were analyzed using PPI, and the resulting network was screened (Fig. **4A**). Two individual clustering modules of closely linked genes were extracted using the MCODE plugin (Fig. **4B**). The MCODE result revealed that Cluster 1 contained five nodes and six edges, whereas Cluster 2 comprised four nodes and six edges. Additionally, network analysis conducted using the CytoHubba plugin with MCC, DMNC, and Degree value as reference criteria identified 16 characteristic shared genes, including TIMP1, IGFBP2, FAP, CTSK, FGF13, VCAN, S100A4, PCOLCE, IGFBP3, LOX, ITGA5, LUM, THBS2, TGFBI, NGF, and IGFBP6 (Fig. **4C**).

### 3.5 SVM-RFE and Random Forest Analysis

**Fig. (5):**
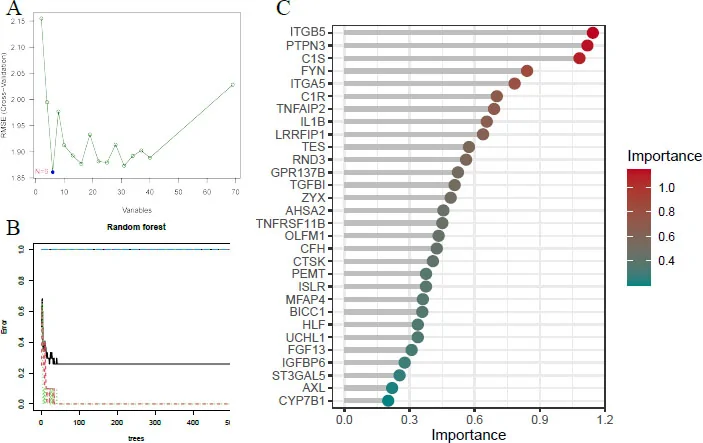
Machine learning analysis. (**A**) SVM-RFE analysis. (**B**) Random forest analysis. (**C**) Characteristic shared genes identified by random forest analysis.

For further analysis, we employed SVM-RFE and random forests. The SVM-RFE analysis identified six genes associated with comorbidities of PE and obesity: UCHL1, CNR1, HLF, ISLR, RGS2, and ISG15 (Fig. (**5A**). In contrast, the random forest analysis revealed three genes: PTPN3, C1S, and ITGB5 (Figs. **5B** and **C**). Both sets of genes were deemed characteristic and demonstrated significant correlations with PE and obesity.

### 3.6 Identification and Analysis of Characteristic Shared Genes

By integrating PPI network analysis with machine learning algorithms, 25 candidate hub genes associated with PE-obesity comorbidity were identified. The genes included TIMP1, IGFBP2, FAP, CTSK, FGF13, VCAN, S100A4, PCOLCE, IGFBP3, LOX, ITGA5, LUM, THBS2, TGFBI, NGF, IGFBP6, UCHL1, CNR1, HLF, ISLR, RGS2, ISG15, PTPN3, C1S, and ITGB5.

**Fig. (6):**
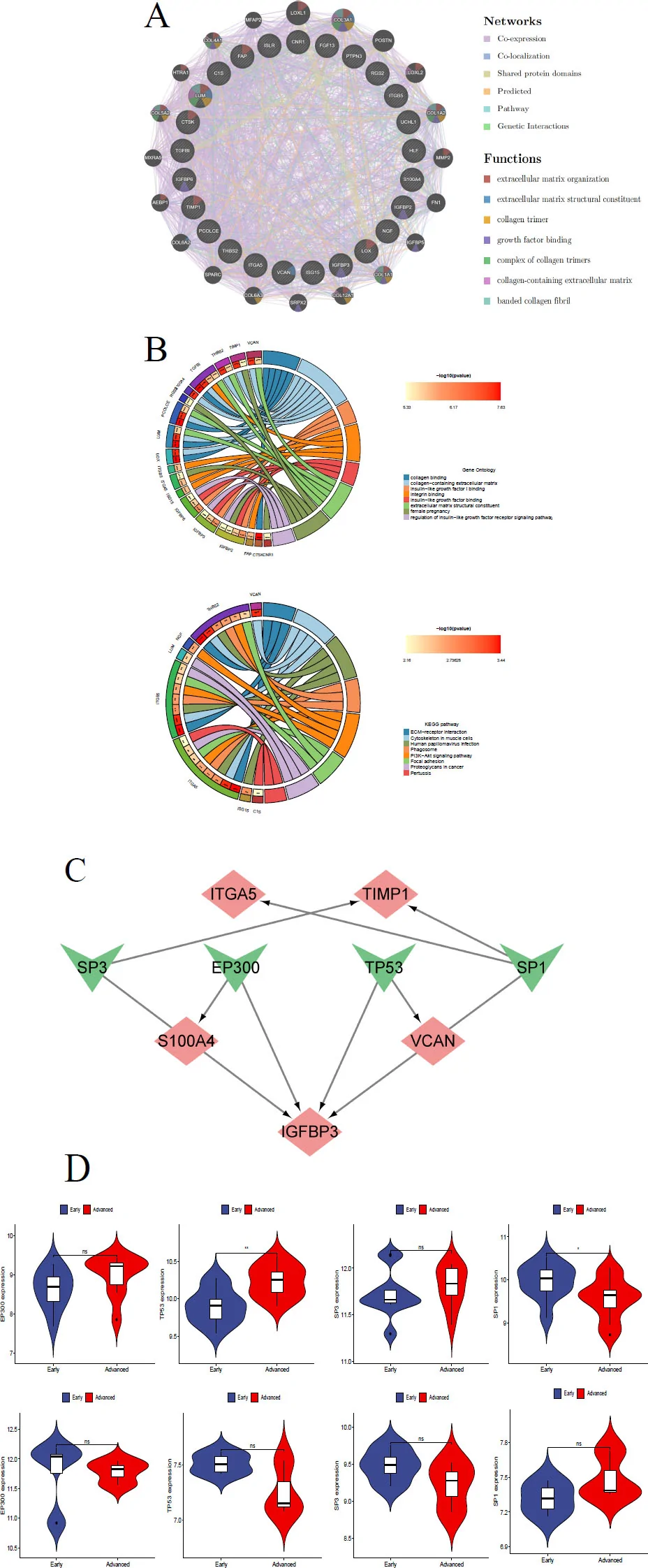
(**A**) Characteristic shared genes, and their co-expression were analyzed using GeneMANIA. (**B**) Circle diagram of GO functional and KEGG enrichment analyses of characteristic shared genes. (**C**) TF regulatory network. TFs are marked in green, and the characteristic shared genes are marked in red. (**D**) Expression levels of TFs in GSE96985 and GSE2508 datasets.

Subsequently, we analyzed the co-expression network and functional roles of the characteristic shared genes using the GeneMANIA database. Network analysis revealed 84.59% co-expression, 7.86% co-localization, 4.65% shared protein domains, 1.73% predicted, 0.63% pathway, and 0.54% genetic interactions (Fig. **6A**).

Enrichment analysis of characteristic shared genes using KEGG identified the top eight pathways, including extracellular matrix-receptor interaction, cytoskeleton in muscle cells, and human papillomavirus infection, which were the primary pathways. ITGA5 and ITGB5 were the genes with the most enriched pathways (Fig. **6B**). GO ontology analysis revealed the top eight processes, including collagen binding, collagen-containing extracellular matrix, and insulin-like growth factor I binding. IGFBP2 and TGFBI were the most enriched genes.

### 3.7 TF Analysis and Construction of Regulatory Network

We found that four TFs regulated the expression of characteristic shared genes using the TRRUST database (Fig. **6C**). Further verification revealed that TP53 and SP1 were differentially expressed in PE (*p* < 0.05; Fig. **6D**). These TFs regulate four genes (IGFBP3, VCAN, ITGA5, and TIMP1).

### 3.8 ROC Curve and Immune Infiltrate Analysis

**Fig. (7):**
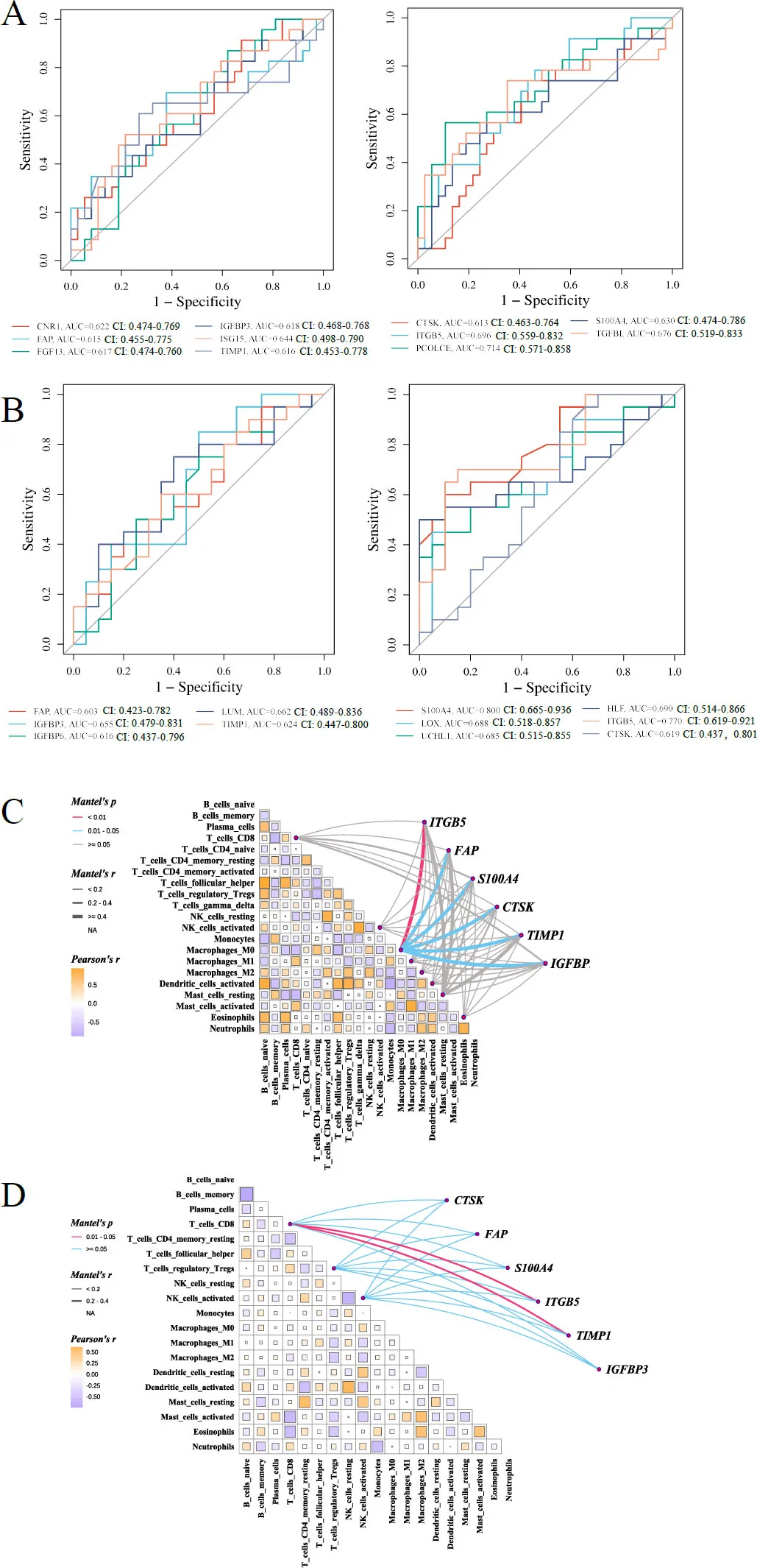
ROC and immune infiltration analyses. (**A**) ROC of characteristic shared genes in PE validation dataset (GSE25906). (**B**) ROC of characteristic shared genes in the obesity validation dataset (GSE15183). (**C**) Correlation matrix and network visualization of gene-immune cell associations in PE. (**D**) Correlation matrix and network visualization of gene-immune cell associations in obesity. The heatmap color represents Pearson's correlation coefficient. Border thickness indicates Mantel's r value. Network lines represent statistically significant associations determined by the Mantel test with permutations: red (*p* < 0.01), blue (0.01 ≤ *p* < 0.05), and gray (*p* ≥ 0.05).

To evaluate the diagnostic potential of the 25 characteristic-shared genes identified from the discovery datasets, we performed ROC curve analysis directly in two independent external validation datasets: GSE25906 (PE cohort) and GSE151839 (obesity cohort). In the PE validation dataset, 11 genes exhibited comparable diagnostic performances (AUC > 0.6). The genes were CNR1, FAP, FGF13, IGFBP3, ISG15, TIMP1, CTSK, ITGB5, PCOLCE, S100A4, and TGFBI (Fig. **7A**). In the obesity validation dataset, 11 other genes exhibited comparable diagnostic performance (AUC > 0.6). The genes were FAP, IGFBP3, IGFBP6, LUM, TIMP1, S100A4, LOX, UCHL1, HLF, ITGB5, and CTSK (Fig. **7B**). Among these, six genes demonstrated consistent diagnostic efficiency in both validation cohorts. The genes, including FAP, IGFBP3, TIMP1, S100A4, ITGB5, and CTSK, were identified as potential robust diagnostic biomarkers for the comorbidity of PE and obesity.

CIBERSORT analysis indicated distinct and shared patterns of immune cell infiltration. In PE-related datasets (Fig. **7C**), FAP, IGFBP3, TIMP1, S100A4, ITGB5, and CTSK were all associated with Macrophages M0, with ITGB5 presenting the strongest correlation (*r* = 0.825, *p* = 0.004). In obesity-related datasets (Fig. **7D**), FAP, IGFBP3, TIMP1, S100A4, ITGB5, and CTSK were primarily associated with CD8+ T cells, regulatory T cells (Tregs), and NK cell activation, among which ITGB5 and TIMP1 exhibited the correlations with CD8+ T cells (*r* = 0.285, *p* = 0.014). CD8+ T cells may represent a common immune cell subset linked to both PE and obesity, and ITGB5 may be associated with this pattern. However, these correlational findings should be interpreted cautiously and require experimental validation.

### 3.9 Validation of Characteristic Shared Genes

**Fig. (8):**
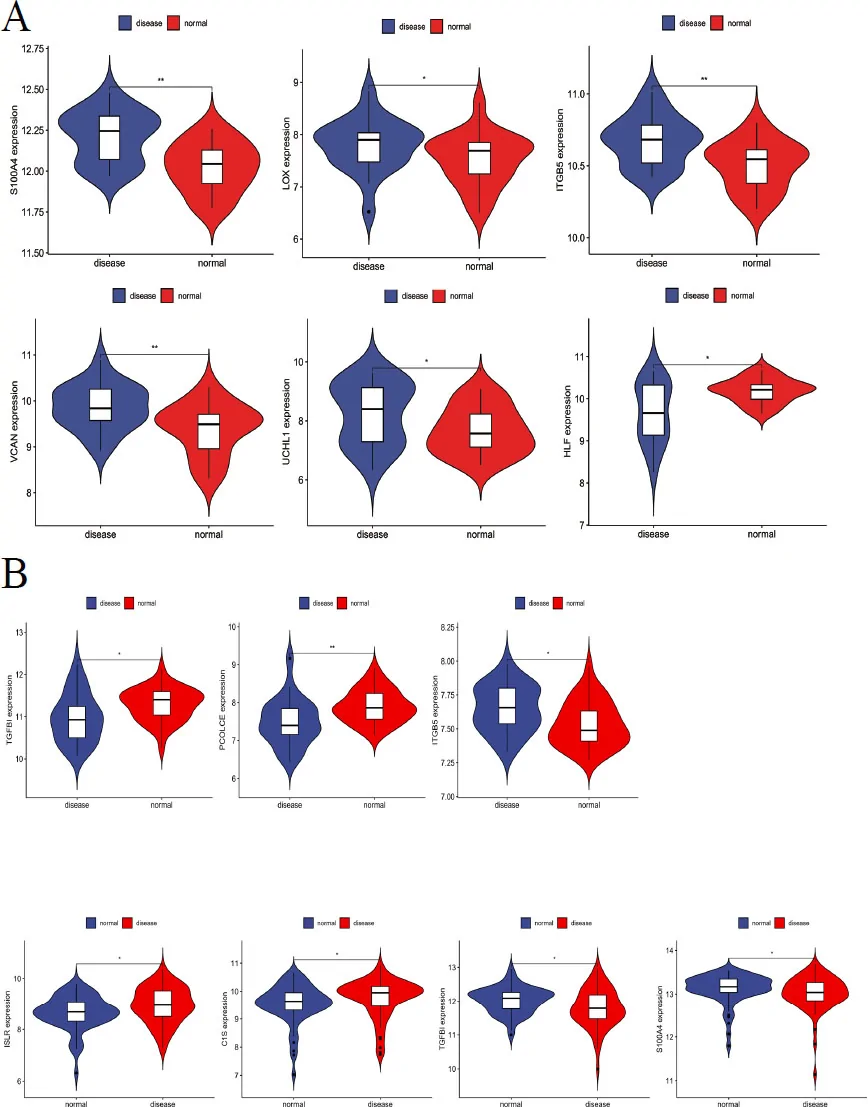
Validation of the characteristic shared gene expression (only the significance level was *p* < 0.05). (**A**) Obesity dataset (GSE151839). (**B**) PE dataset (GSE60438 and GSE25906).

We conducted a validation analysis of the characteristic shared genes associated with the PE and obesity comorbidity, as identified in the preceding analysis. This analysis used PE-related datasets GSE60438 and GSE25906, as well as the obesity-related dataset GSE151839. The significance level was *p* < 0.05. The results revealed that three characteristic genes were differentially expressed in the PE-related dataset GSE60438 (TGFBI, PCOLCE, and ITGB5) and four in dataset GSE25906 (ISLR, C1S, TGFBI, and S100A4). In the context of obesity, six characteristic genes were identified: S100A4, PCOLCE, UCHL1, HLF, VCAN, and ITGB5, all of which exhibited statistically significant differences. ITGB5 emerged as the sole characteristic gene demonstrating consistency across the two validation datasets (Fig. **8**). Based on the results of these analyses, ITGB5 was selected for further investigation using single-gene GSEA and drug forecasting.

### 3.10 GSEA and Human Protein Atlas Database Analysis

**Fig. (9):**
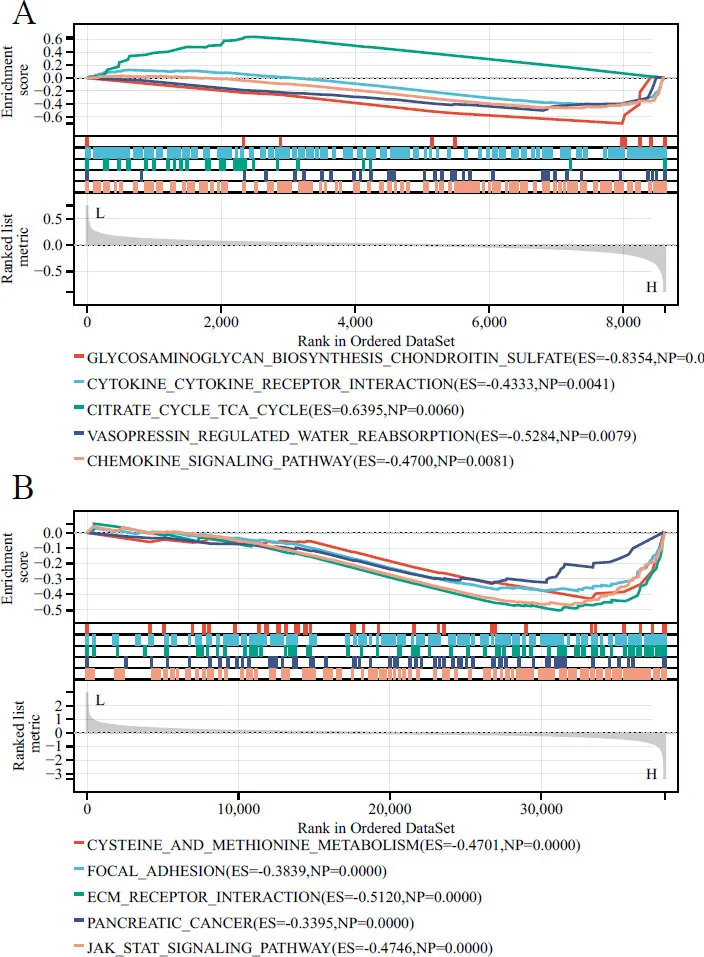
GSEA enrichment analysis. (**A**) Obesity dataset (GSE96985). (**B**) PE dataset (GSE2508).

Based on the GSEA findings, in the context of obesity, ITGB5 was primarily associated with the following pathways: glycosaminoglycan biosynthesis, chondroitin sulfate, cytokine receptor interaction, citrate cycle, vasopressin-regulated water reabsorption, and chemokine signaling pathway (top five). PE was associated with cysteine and methionine metabolism, focal adhesion, ECM receptor interaction, pancreatic cancer, and JAK-STAT signaling pathway (top five) (Fig. **9**).

**Fig. (10):**
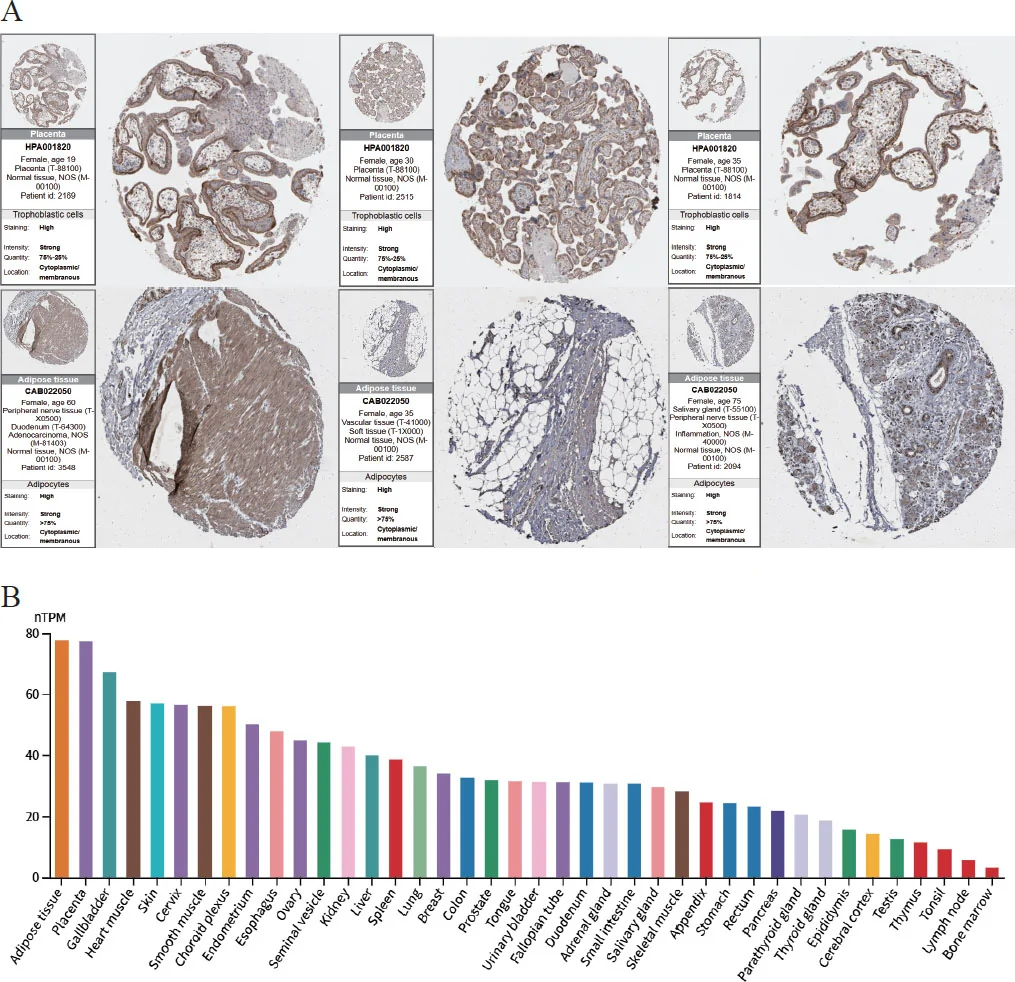
ITGB5 expression in human placenta and adipose tissue (HPA database). (**A**) IHC staining of ITGB5 in the placenta and adipose tissue. (**B**) ITGB5 RNA-seq expression (nTPM) in normal tissues: adipose tissue and placenta exhibited the highest expression among all detected tissues.

These findings suggest that ITGB5 may be a candidate gene involved in both PE and obesity, with a consistent association with immune cell infiltration, supporting its potential diagnostic relevance. HPA database verification revealed that ITGB5 is highly expressed in the human placenta and adipose tissue at both protein and RNA levels. At the protein level, IHC staining showed strong intensity, positive signals in >75% of cells, and cytoplasmic or membranous localization. At the RNA level, RNA-seq data demonstrated that ITGB5 expression in the placenta and adipose tissue was significantly higher than in other normal tissues, ranking among the top two across all detected tissues (Fig. **10**). This dual-tissue high-expression pattern confirms that ITGB5 has a tissue basis and participates in the pathological processes of both diseases, further supporting its pathogenicity and functional validity.

### 3.11 Drug Forecasting and Analysis

**Fig. (11):**
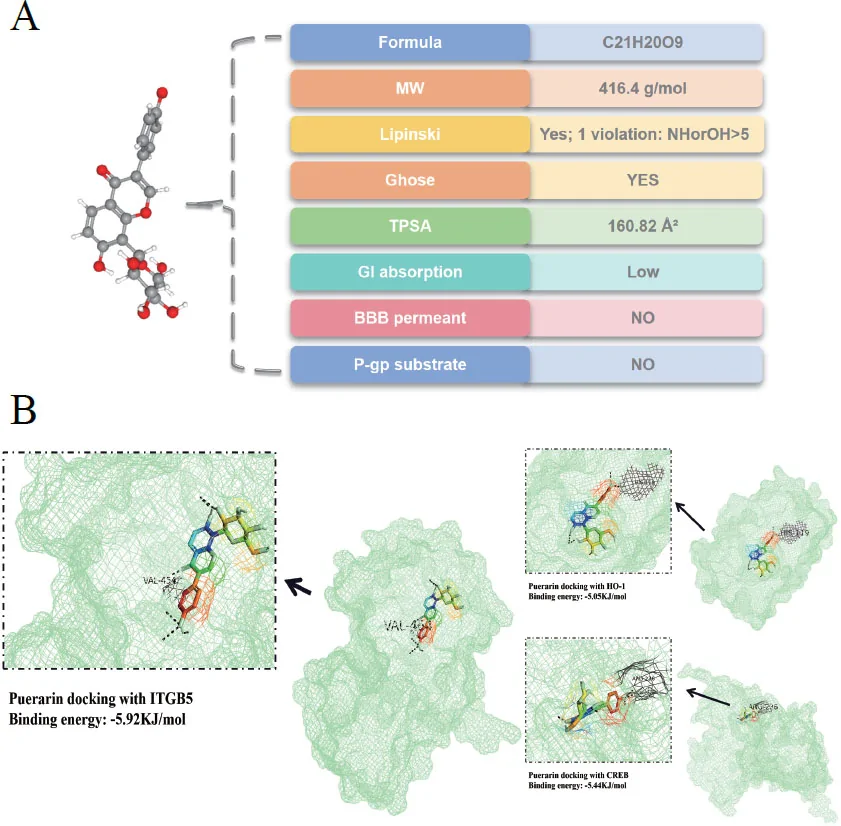
Drug forecasting and molecular docking analysis (**A**) ADMET of puerarin. (**B**) Docking of ITGB5, HO-1, and CREB (as controls) with puerarin.

In this study, we employed HIT 2.0 to identify potential TCM small molecules that interact with the key target protein ITGB5. The results revealed that puerarin was the only TCM small molecule targeted by ITGB5. According to existing literature, puerarin has been reported to treat both PE and obesity [16, 17]. Therefore, puerarin has emerged as a potential target for TCM small molecules. SwissADME analysis revealed that puerarin complied with Lipinski's Rule of Five but exhibited low gastrointestinal absorption. The results demonstrated that puerarin is a promising therapeutic compound. Subsequently, we performed molecular docking analysis. Based on reports indicating that puerarin alleviates PE symptoms through the CREB/HO-1 pathway [18], we selected CREB and HO-1 as reference targets to evaluate docking scores. Binding energy calculations revealed that puerarin bound to ITGB5 (binding energy: –5.92 kJ/mol) with an affinity comparable to that of CREB (binding energy: –5.44 kJ/mol) and HO-1 (binding energy: –5.05 kJ/mol), indicating that the three protein targets exhibit similar binding potential for this compound (Fig. **11**).

**Fig. (12):**
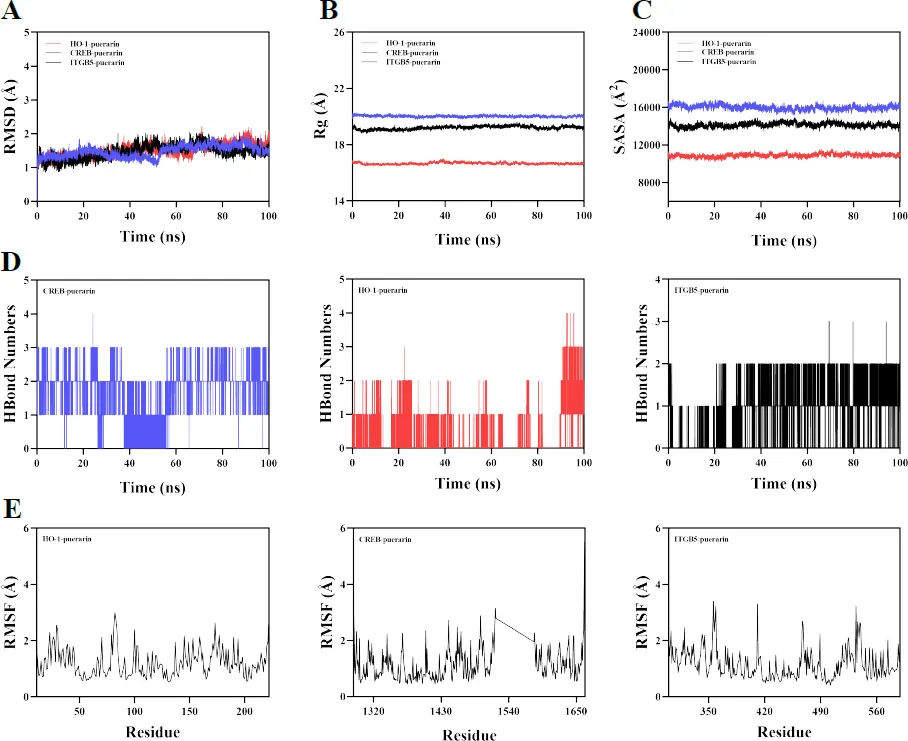
Molecular dynamics simulation of the complex formed between the protein and its ligand. (**A**) Time-dependent changes in RMSD of the complex. (**B**) Time-dependent changes in the radius of gyration (Rg). (**C**) Time-dependent changes in solvent-accessible surface area (SASA). (**D**) Time-dependent changes in the number of hydrogen bonds (HBonds). (**E**) Per-residue root-mean-square fluctuation (RMSF) across the complex.

RMSD is a reliable indicator for assessing the conformational stability of proteins and ligands, reflecting the degree of deviation of atomic positions from their initial coordinates. As shown in Fig. (**12A**), the puerarin small molecule exhibited high stability when bound to HO-1, CREB, and ITGB5. The radius of Rg indicated that the HO-1–puerarin, CREB–puerarin, and ITGB5–puerarin complexes maintained relatively stable fluctuations during simulation (Fig. **12B**). SASA analysis revealed no significant changes in the complexes following binding, further suggesting stable interactions between the protein targets and the small molecule (Fig. **12C**). The number of hydrogen bonds formed between puerarin and each target protein during the simulation is shown in Fig. (**12D**). For HO-1–puerarin, hydrogen bond numbers ranged from 0 to 4, with one hydrogen bond most frequently observed. For CREB–puerarin, hydrogen bond numbers ranged from 0 to 4, with one to two hydrogen bonds most frequently observed. For ITGB5–puerarin, hydrogen bond numbers ranged from 0 to 3, with one to two hydrogen bonds most frequently observed. These results indicate a sustained yet dynamic hydrogen bonding interaction. RMSF results are presented in Fig. (**12E**). The RMSF values for the HO-1–puerarin, CREB–puerarin, and ITGB5–puerarin complexes were relatively low, indicating limited flexibility and high structural stability. In summary, puerarin exhibited comparable binding affinities for HO-1, CREB, and ITGB5, indicating similar binding potential between the ITGB5 and CREB/HO-1, both of which have been identified as therapeutic targets of puerarin in preeclampsia in previous studies.

## 4 DISCUSSION

PE and obesity exhibit significant pathophysiological overlap, particularly in terms of shared inflammatory responses, immune-metabolic modulation, and oxidative stress [19]. Chronic inflammation, driven by adipose tissue-derived cytokines, exacerbates placental dysfunction, while the immune-metabolic disturbances associated with obesity mirror the angiogenic imbalance observed in PE [20, 21]. Despite these observations, the shared pathogenic mechanisms linking obesity and PE remain inadequately understood. Our bioinformatics analysis identifies key molecular mediators of this comorbidity from a novel perspective and predicts potential therapeutic agents, such as anti-inflammatory and immune-metabolic modulation herbal monomers. These agents may offer therapeutic benefits for PE in the context of obesity during pregnancy by concurrently targeting these interconnected pathways.

### 4.1 Inflammation: A Central Link Between Obesity and PE

Chronic low-grade inflammation is a hallmark of obesity and PE. In obesity, excessive accumulation of adipose tissue, particularly visceral fat, secretes substantial amounts of pro-inflammatory factors that directly impair vascular endothelial function and activate signaling pathways, such as Nuclear Factor kappa-B (NF-κB) [22]. In this study, we identified several genes characteristic of both PE and obesity, including FAP, IGFBP3, TIMP1, S100A4, ITGB5, and CTSK. Among these, cathepsin K (CTSK), a lysosomal cysteine protease, is closely associated with chronic low-grade inflammatory states. In obesity, excessive accumulation of adipose tissue, particularly visceral fat, secretes a substantial amount of pro-inflammatory factors. These factors directly impair vascular endothelial function and may activate signaling pathways such as NF-κB. Research indicates that CTSK can facilitate the release of pro-inflammatory cytokines, such as TNF-α and IL-6, by activating inflammatory signaling pathways, including NF-κB, thereby leading to hemodynamic disturbances [23, 24]. Besides its role in inflammation, CTSK has been observed to be significantly elevated in adipose tissue from obese individuals and in animal models fed an HFD [24]. CTSK plays a significant role in metabolic dysregulation by influencing adipocyte differentiation and function. Experimental studies have demonstrated that CTSK inhibitors can effectively reduce adipose tissue hypertrophy and improve insulin resistance in mice, suggesting their potential as a therapeutic target for obesity [25]. The regulatory effects of CTSK on the immune microenvironment may also influence macrophage function. Recent research indicates that CTSK can modulate macrophage polarization by affecting the TLR4/MyD88 signaling pathway, thereby promoting pro-inflammatory M1 polarization and inhibiting anti-inflammatory M2 polarization. This mechanism may directly contribute to metabolic inflammation and insulin resistance associated with obesity [26]. In PE pathogenesis, hyperactivated inflammatory responses and immune imbalance are central pathological features.

Beyond their effects on maternal vascular function, these inflammatory mediators may also directly impact fetal development [27]. Emerging evidence suggests that maternal inflammation, particularly when driven by obesity, disrupts placental nutrient transport and impairs fetal growth trajectories [28]. Elevated maternal IL-6 levels have been associated with reduced fetal amino acid supply *via* downregulation of placental System A amino acid transporters, contributing to intrauterine growth restriction in obese pregnancies [29]. These findings align with our observation that CTSK and S100A4, both involved in NF-κB activation and pro-inflammatory cytokine release [30, 31], may contribute to maternal vascular dysfunction and adverse fetal outcomes. Furthermore, chronic inflammation has been shown to alter placental DNA methylation patterns at imprinted genes critical for fetal growth, such as IGF2 and H19 [32], providing an epigenetic link between maternal obesity, inflammation, and lifelong metabolic programming in offspring.

### 4.2 Oxidative Stress: A Shared Pathological Mechanism

Oxidative stress is another critical intersection between obesity and PE. In this study, S100A4 was identified as a gene that promotes inflammation, enhances oxidative stress, and induces apoptosis. S100A4, a pro-inflammatory and pro-fibrotic calcium-binding protein, exacerbates inflammatory responses and facilitates macrophage infiltration [33]. Obesity-related insulin resistance and hyperleptinemia may enhance the biological effects of S100A4 through pathways such as JAK/STAT and mTOR [34, 35]. Simultaneously, elevated levels of S100A4 can diminish cellular invasive capacity while promoting oxidative stress and apoptosis [36, 37], potentially exacerbating abnormal migration of vascular smooth muscle cells and impairing the remodeling of placental spiral arteries. These effects may lead to placental ischemia-hypoxia, ultimately resulting in the characteristic pathological changes observed in patients with PE. The identification of puerarin as a potential therapeutic agent further supports the role of oxidative stress in this comorbidity. Puerarin has demonstrated significant antioxidant properties by inhibiting oxidative stress-related signaling pathways, mitigating reactive oxygen species production, and enhancing antioxidant enzyme activity [38]. In PE-obesity comorbidity, where enhanced oxidative stress damages placental and vascular tissues, the antioxidant mechanism of puerarin may offer therapeutic benefits.

### 4.3 Immune Modulation: Dysregulation of CD8+ T Cells and Macrophage Polarization

Our analysis suggests that CD8+ T cells may represent a shared immune-related response in obesity and PE, although this finding requires further validation. Current research suggests that CD8+ T cell immunity plays a complex and pivotal role in both conditions. Shared mechanisms can be explored from the functional imbalance of immune cells and the interaction between metabolism and immunity. Regarding cell functional imbalance, the immune regulation of CD8+ T cells in decidual tissue is disrupted in patients with PE. Studies have demonstrated that individuals with early-onset PE exhibit a significant reduction in decidual Treg cells and an increased proportion of CD8+ T cells [39]. This imbalance disrupts the normal immune cell equilibrium during pregnancy, as Treg cells are unable to adequately suppress the cytotoxic activity of CD8+ T cells, thereby shifting the immune response towards attacking fetal antigens. This disruption may result in vascular and trophoblastic damage to the placenta, ultimately impairing placental function. In obesity-related pathologies, such as non-alcoholic fatty liver disease, both the quantity and functionality of CD8+ T cells in the hepatic environment undergo significant alterations. In obesity and hyperlipidemia, these cells can activate hepatic stellate cells, thereby promoting inflammation and fibrosis [40]. This observation indicates that obesity disrupts the normal functioning of CD8+ T cells, and when it coexists with PE, it may further exacerbate immune cell dysfunction, thereby aggravating disease progression. Regarding the interplay between metabolism and the immune system, obesity induces systemic metabolic disturbances that substantially affect CD8+ T cell immune function. Research in tumor biology has demonstrated that in obese murine models, the infiltration and functionality of CD8+ T cells within tumors are suppressed, a phenomenon linked to reduced chemokine expression in the tumor microenvironment and impaired amino acid metabolism [41]. Similarly, in obesity and PE comorbidity, metabolic abnormalities may compromise CD8+ T cell function. Metabolic alterations induced by obesity may lead to the depletion of essential nutrients, such as glutamine, which are crucial for the functional capacity of CD8+ T cells [42]. In individuals with PE, preexisting conditions, such as placental ischemia and hypoxia, can further exacerbate these metabolic disruptions, thereby impairing the immune functionality of CD8+ T cells and perpetuating a detrimental cycle that worsens the disease. Additionally, the accumulation of metabolic byproducts associated with obesity may trigger inflammatory responses, maintaining CD8+ T cells in a prolonged state of stress and compromising their normal immunoregulatory functions [43]. In PE, this effect may be magnified, increasing the risk to maternal and fetal health.

In this study, we found that ITGB5 was correlated with CD8+ T cells in the obesity dataset GSE151839 (*r* = 0.285, *p* = 0.014), while in the PE dataset GSE25906, it was strongly correlated with Macrophages M0 (*r* = 0.825, *p* = 0.004). These results suggest that CD8+ T cells may serve as a shared immune feature across both conditions, particularly in the context of obesity. The potential involvement of CD8+ T cells in PE should be interpreted within the framework of maternal-fetal immune tolerance, acknowledging that our findings are correlational and require further validation. During normal pregnancy, decidual CD8+ T cells exhibit an exhausted phenotype with reduced cytotoxic potential, thereby protecting the trophoblast from immune attack [44]. However, this balance is disrupted in PE. These finding of increased proportions of CD8+ T cells aligns with research reporting reduced decidual Treg cell numbers and increased CD8+ T cell numbers in early-onset PE [45]. Recent studies have confirmed these observations. Research has revealed that decidual CD8+ T cells from patients with PE display an activated effector memory phenotype, with upregulated granzyme B and perforin [46]. Another study demonstrated impaired PD-1/PD-L1 checkpoint signaling in preeclamptic decidua, leading to inadequate suppression of CD8+ T cell activity [47]. Obesity-related metabolic disturbances may exacerbate immune alterations. Obesity-induced leptin resistance and hyperinsulinemia promote pro-inflammatory CD8+ T cell responses while suppressing Treg differentiation [48]. Additionally, macrophages play an essential role in mediating CD8+ T cell inflammation during obesity. Obesity induces PD-1 expression on tumor-associated macrophages, thereby suppressing T cell stimulatory potential and contributing to a pro-inflammatory environment [49]. This is complemented by findings that macrophages mediate increased CD8+ T cell inflammation during weight loss in formerly obese mice, indicating a persistent inflammatory state despite improvements in metabolic profiles [50]. During pregnancy, these metabolic effects may shift the decidual immune balance further toward rejection rather than tolerance, providing a mechanistic framework for understanding how obesity increases the risk of PE.

### 4.4 ITGB5: Candidate Role in PE-obesity Comorbidity

Our validation studies, by identifying characteristic genes with high diagnostic performance, demonstrated that the ITGB5 gene consistently exhibits expression patterns across multiple datasets, suggesting it may be a co-expressed gene common to both obesity and PE. Meanwhile, we verified in the HPA database that ITGB5 was highly expressed at both the protein and RNA levels in the placenta, the core pathological tissue of PE, and in adipose tissue, the characteristic tissue of obesity. In the placenta, ITGB5 exhibited strong, extensive expression with cytoplasmic or membranous localization in > 75% of positive cells, providing direct evidence of tissue expression and supporting its involvement in placental-related biological functions, suggesting its potential role in the pathological progression of PE by regulating placental tissue function. To understand the role of ITGB5 in PE, it is essential to investigate its impact on trophoblast cell function. As an integrin protein involved in cell adhesion and signal transduction, ITGB5 expression alteration during PE may significantly influence the biological behavior of trophoblast cells. ITGB5 expression may be regulated by multiple molecular mechanisms. Studies have demonstrated that miR-134 suppresses the invasive capacity of trophoblast cells by downregulating ITGB1 expression, and ITGB5 may play a role in a similar regulatory network [51]. This microRNA-mediated regulatory mechanism highlights the complexity of ITGB5 in the pathophysiology of PE. Other studies have also revealed signaling pathways associated with ITGB5 function. Abnormal expression and methylation status of FN1, FOS, and ITGA5 may promote the development of PE by regulating trophoblast differentiation, apoptosis, and invasion [52]. These findings highlight the potential role of ITGB5 in PE and suggest its therapeutic potential. Regarding the role of ITGB5 in obesity, studies have demonstrated that ITGB5 is essential for regulating ECM-cell adhesion, a function closely associated with metabolic disorders related to obesity [53]. Additionally, ITGB5 modulates high-glucose-induced endothelial cell apoptosis by regulating the FoxO1-mediated autophagy pathway, thereby influencing diabetes-related vascular complications [54]. In the pathophysiology of obesity, ITGB5 regulates lipid storage and metabolism in intestinal epithelial cells by interacting with its ligand fibronectin [55]. This mechanism suggests that ITGB5 may indirectly influence the development and progression of obesity by modulating the intestinal lipid processing capacity. Further studies have revealed that ITGB5 plays a significant role in regulating immune cell infiltration and polarization, which is closely associated with the chronic, low-grade inflammatory state associated with obesity [56]. In obesity, ITGB5 may influence the inflammatory response in adipose tissue by modulating immune cell behavior, thereby exacerbating insulin resistance and metabolic disorders [57]. ITGB5 emerged as a candidate gene potentially linking PE and obesity, with consistent expression across datasets and high expression confirmed in the placenta and adipose tissue. Its role in this comorbidity may involve regulation of trophoblast function, modulation of immune cells, and metabolic processes, suggesting shared mechanisms of inflammation, immune dysregulation, and metabolic dysfunction.

### 4.5 Potential Therapeutic Implications: Puerarin as a Multi-target Agent

Subsequent analyses identified puerarin as a potential therapeutic agent. Puerarin, a natural medicinal compound derived from the traditional Chinese herb kudzu root, has been extensively studied for its multifaceted potential to address both PE and obesity. A thorough review of existing studies indicates that the therapeutic mechanism of puerarin in managing the comorbidity of PE and obesity may involve critical processes, including the inhibition of inflammation and oxidative stress and the protection of vascular endothelium. Regarding the inhibition of inflammation and oxidative stress, extensive research has demonstrated significant anti-inflammatory and antioxidant properties of puerarin [58-60]. Specifically, puerarin has been demonstrated to downregulate the expression of inflammatory factors, such as TNF and IL-6, induced by lipopolysaccharide stimulation in PE cell models [15]. In obesity-related research, puerarin has been observed to reduce macrophage infiltration in adipose tissue of obese mice, decrease the expression of inflammatory factors such as TNF-α, and enhance the inflammatory microenvironment [16]. In the framework of PE and obesity comorbidity, inflammatory responses are acknowledged as pivotal contributors to disease progression. The inhibition of these responses by puerarin suggests its potential as a promising therapeutic strategy. Additionally, puerarin exhibited antioxidant properties in cardiovascular disease by inhibiting oxidative stress-related signaling pathways [61]. In PE and obesity comorbidity, enhanced oxidative stress damages cells and tissues. Puerarin potentially mitigated reactive oxygen species production, enhanced antioxidant enzyme activity, and alleviated oxidative damage *via* its antioxidant mechanisms, thereby ameliorating associated comorbidities. These findings are consistent with our hypothesized common pathogenic mechanisms involving the ITGB5 target in both obesity and PE. Additionally, research has demonstrated that puerarin protects vascular endothelial cells. It facilitates the proliferation, migration, and tube formation of these cells, thereby enhancing their activity and promoting angiogenesis *via* mechanisms such as autophagy activation [54]. PE is characterized by vascular endothelial dysfunction, which is exacerbated by obesity. Endothelial protective properties of puerarin contribute to the maintenance of normal vascular function, improving both placental and systemic blood circulation and positively impacting the management of this comorbidity. Additionally, in obese mouse models, puerarin has been observed to improve lipid metabolism, indirectly safeguarding the vascular endothelium by mitigating dyslipidemia-associated damage [62]. Puerarin has emerged as a potential therapeutic agent that targets the shared pathogenic mechanisms of PE and obesity. Its anti-inflammatory, antioxidant, and endothelial protective properties align with the proposed role of ITGB5 in this comorbidity, suggesting that puerarin may exert its effects by modulating ITGB5-related pathways.

### 4.6 Study Innovations and Limitations

This study presents an innovative theoretical framework for understanding the comorbidity between PE and obesity and identifies potential therapeutic targets through integrated multi-omics bioinformatics and MR [63]. By moving beyond examining these conditions in isolation, we systematically uncovered shared molecular interactions and causal relationships, offering new insights into their interconnected pathophysiology. Nevertheless, several limitations of this study should be addressed. First, the public datasets used were derived from diverse platforms and populations, potentially introducing batch effects despite cross-dataset validation. Second, their cross-sectional nature lacks temporal information, precluding causal inference or tracking of disease progression. Third, reliance on public expression profiles means limited sample sizes and incomplete clinical annotation. Fourth, this study lacks functional validation of ITGB5 (*e.g.*, qPCR, siRNA, tissue staining). The HPA data included are descriptive protein expression evidence only and do not constitute functional validation. Future experimental studies are essential to confirm our findings. Similarly, our identification of CD8+ T cells as a potential shared immune axis between obesity and PE is based solely on CIBERSORT correlation analyses and has not been validated by experimental methods such as flow cytometry, immunohistochemistry, or functional assays. These findings should be interpreted as hypothesis-generating. This remains a bioinformatics-driven study without functional validation. Although MR supports causality, the roles of hub genes, such as ITGB5, CTSK, and S100A4, require experimental confirmation *via* *in vitro* and *in vivo* studies. The diagnostic performance of these gene signatures also needs to be prospectively validated in independent cohorts. Future research should focus on (i) functional validation of characteristic genes, (ii) longitudinal cohort studies, (iii) prospective evaluation of diagnostic models, and (iv) translational studies on puerarin. Addressing these gaps will help develop personalized strategies for high-risk pregnancies.

## CONCLUSION

In summary, this study identified several genes (FAP, IGFBP3, TIMP1, S100A4, ITGB5, and CTSK) associated with both PE and obesity. ITGB5 emerged as a candidate gene that correlated with Macrophages M0 in PE and with CD8+ T cells in obesity, suggesting a potential role in immune–metabolic dysregulation. Given the bioinformatic nature of this study, these findings are hypothesis-generating and require experimental validation. Bioinformatics also predicted puerarin as a candidate therapeutic agent, pending functional testing. Overall, this study provides preliminary insights into shared mechanisms and highlights the utility of bioinformatics for drug discovery. Further functional and clinical studies are needed.

## Figures and Tables

**Fig. (2):**
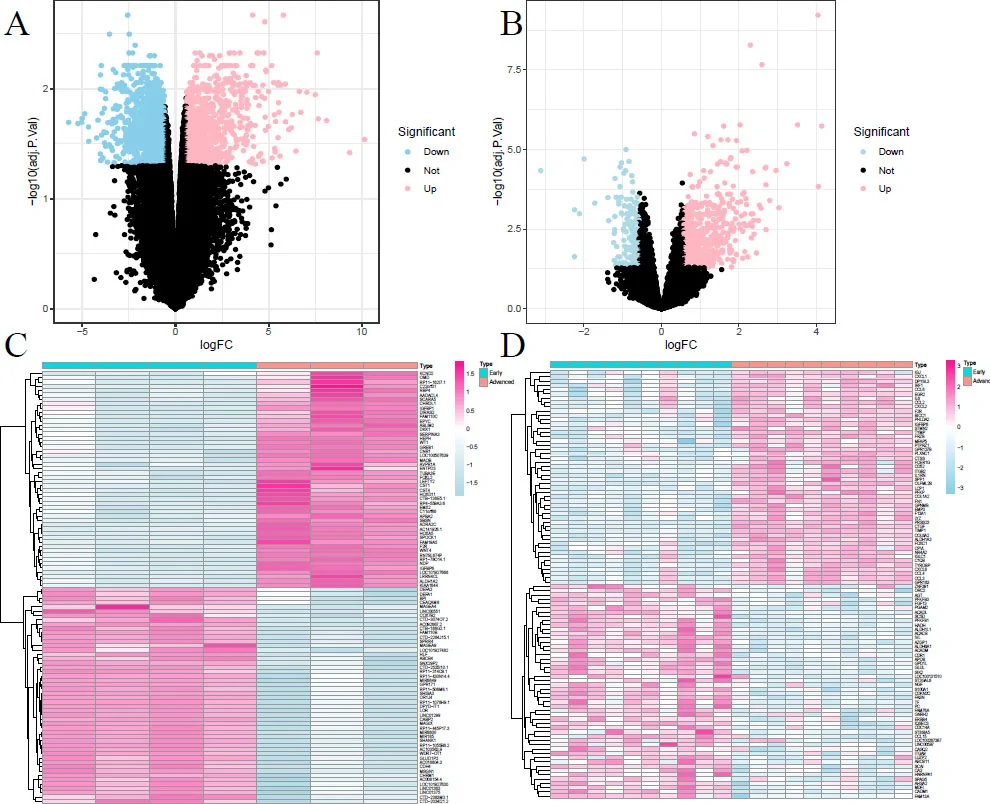
DEGs in PE and obesity. (**A**) Volcano plot of DEGs in the PE dataset, with red indicating upregulated genes and blue indicating downregulated genes. (**B**) Volcano plot of DEGs in the obesity dataset, with red indicating upregulated genes and blue indicating downregulated genes. (**C**) Heatmap of DEGs in the PE dataset. Red indicates high expression, and blue indicates low expression. (**D**) Heatmap of DEGs in the obesity dataset. Red indicates high expression, and blue indicates low expression.

**Fig. (3):**
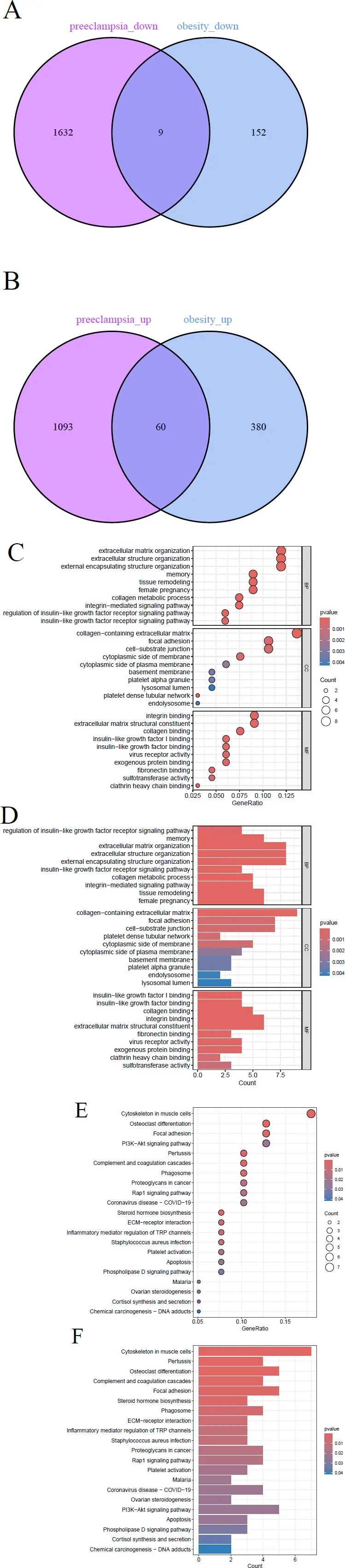
Screening for genes shared between PE and obesity comorbidity. (A) Intersection of downregulated DEGs in PE and obesity. Red indicates the PE dataset, and blue indicates the obesity dataset. (**B**) Intersection of upregulated DEGs in PE and obesity. Red indicates the PE dataset, and blue indicates the obesity dataset. (**C**) Bubble diagram of GO functional analysis. (**D**) Histogram of GO functional analysis. (**E**) Bubble diagram of KEGG enrichment analysis. (**F**) Histogram of KEGG enrichment analysis.

**Table 1:** Characteristics of GWAS datasets and MR analysis parameters.

**Parameter**	**Obesity (Exposure)**	**PE (Outcome)**
GWAS ID	ieu-a-90	ukb-b-13535
Author	Berndt SI, 2013	Ben Elsworth, 2018
Population	European	European
Sample size	98697	462933
Cases/Controls	32,858/65,839	1,864/461,069
Total SNPs	2380428	9851867
IV selection threshold	*p* < 5 × 10^–5^	
LD clumping (r^2^/kb)	0.001/10,000	
Final IVs for MR	100	
Mean F-statistic	10	
Cochran’s Q/Q_*p* value	75.851/0.296	
MR-Egger Intercept/*p* value	–5.729 × 10^−5^/0.262	
MR-PRESSO/*p* value	79.191/0.314	

**Table 2:** Specific information on the source of the data.

**Disease**	**GEO accession**	**Platform**	**Group**
PE (analysis)	GSE96985	GPL22120	8 samples for normal and 6 samples for PE
PE (validation)	GSE60438	GPL6884	9 samples for normal and 9 samples for PE
PE (validation)	GSE25906	GPL6102	37 samples for normal and 23 samples for PE
Obesity (analysis)	GSE2508	GPL91	10 samples for lean and 10 samples for obesity
Obesity (validation)	GSE151839	GPL570	20 samples for obese and 20 samples for normal weight

## Data Availability

The data used in this study will be available from the corresponding author upon request. The datasets presented in this study are available in GEO (https://www.ncbi.nlm.nih.gov/geo/). GSE96985, GSE604 38, GSE25906, GSE2508, and GSE151839 datasets were downloaded from the GEO database.

## LIST OF ABBREVIATIONS

PE: Preeclampsia
BMI: Body Mass Index
TNF-α: Tumor Necrosis Factor-alpha
IL-6: interleukin-6
HFD: High-Fat Diet
GEO: Gene Expression Omnibus
MR: Mendelian Randomization
GWAS: Genome-Wide Association Studies
SNPs: Single-Nucleotide Polymorphisms
IVs: Instrumental Variables
LD: Linkage Disequilibrium
DEGs: Differentially Expressed Genes
FDR: False Discovery Rate
FC: Fold Change
GO: Gene Ontology
KEGG: Kyoto Encyclopedia of Genes and Genomes
BP: Biological Process
MF: Molecular Function
CC: Cellular Component
PPI: Protein-protein Interaction
SVM-RFE: Support Vector Machine-Recursive Feature Elimination
TFs: Transcription factors
ROC: Receiver Operating Characteristic Curve
AUC: An area Under the Curve
CIs: Confidence Intervals
GSEA: Gene Set Enrichment Analysis
HPA: Human Protein Atlas
IHC: Immunohistochemistry
ITGB5: integrin β5
TCM: Traditional Chinese Medicine
NF-κB: Nuclear Factor kappa-B
CTSK: Cathepsin K

## STANDARDS OF REPORTING

STROBE-MR guidelines and methodology were followed.
